# Dynamics of HMBG1 (High Mobility Group Box 1) during radiochemotherapy correlate with outcome of HNSCC patients

**DOI:** 10.1007/s00066-021-01860-8

**Published:** 2021-10-20

**Authors:** Kerstin Clasen, Stefan Welz, Heidrun Faltin, Daniel Zips, Franziska Eckert

**Affiliations:** 1 Department of Radiation Oncology, Medical Faculty and University Hospital, Eberhard Karls University, Hoppe-Seyler-Straße 3, 72076 Tuebingen, Germany; 2 German Cancer Research Center (DKFZ) partner site Tuebingen, German Cancer Consortium (DKTK), Hoppe-Seyler-Straße 3, 72076 Tuebingen, Germany; 3 Section for Experimental Radiation Oncology, Department of Radiation Oncology, Medical Faculty and University Hospital, Eberhard Karls University, Hoppe-Seyler-Straße 3, 72076 Tuebingen, Germany

**Keywords:** Immunogenic cell death, Head and neck cancer, Plasma, Biomarker, Radiotherapy

## Abstract

**Purpose:**

High Mobility Group Box 1 (HMGB1) protein has been described as a consensus marker for immunogenic cell death (ICD) in cancer. To personalize treatments, there is a need for biomarkers to adapt dose prescription, concomitant chemotherapy, and follow-up in radiation oncology. Thus, we investigated the levels of HMGB1 in plasma of patients with head and neck squamous cell carcinoma (HNSCC) during the course of radiochemotherapy and follow-up in correlation with oncologic outcome and clinical confounders.

**Methods:**

In our pilot study, 11 patients with advanced HNSCC were treated with definitive radiochemotherapy. Blood samples were taken weekly during treatment and frequently at follow-up visits. HMGB1 levels as well as routine laboratory values were measured and clinical information was collected including tumor volume, infections, toxicity, and follow-up data.

**Results:**

In total, 85 samples were analyzed. In eight patients, HMGB1 levels (baseline vs. last available sample during treatment) were increasing and in three patients HMGB1 values were decreasing toward the end of treatment. All three patients with decreasing values developed tumor recurrence. By contrast, no relapse occurred in patients that showed increasing HMGB1 levels during therapy. Moreover, a positive correlation of HMGB1 levels with tumor volumes, C‑reactive protein (CRP) levels, infections, and grade three toxicity (RTOG) was observed.

**Conclusion:**

HMGB1 might be a promising marker to monitor ICD in HNSCC during the course of radiochemotherapy. However, HMGB1 seems to reflect complex and diverse immunogenic responses and potential confounders. Infections and treatment-associated toxicity should be considered when interpreting the dynamics of HMGB1.

## Introduction

For patients with locally advanced head and neck squamous cell carcinoma (HNSCC), definitive radiochemotherapy is a standard treatment. However, in spite of intense treatment, tumor recurrence remains an issue since locoregional control rates of only approximately 60% after 2 years are reported [1]. Therefore, there is a need for personalized therapeutic approaches and the identification of biomarkers to individualize therapy (de-)escalation during the course of radiotherapy [2–4]. For this purpose, blood-based biomarkers seem promising as they are easy to obtain and repeatedly achievable during treatment. Recently, increasing attention has been paid to immune-related markers to monitor cancer treatment and response.

High Mobility Group Box 1 (HMGB1) protein has been described to act as both a chromatin associated, non-histone transcription factor in the nucleus [5, 6] as well as a mediator of immune response if released to the extracellular space [7–9]. The latter might be of clinical use as a biomarker since increased serum levels of HMBG1 were found in a “late” mediator of immune response 8 h after endotoxin stimulation in a murine model and were proven to be elevated in patients with sepsis [7]. HMGB1 specifically stimulates tumor necrosis factor (TNF) synthesis in monocytes as well as the synthesis of diverse further proinflammatory cytokines as downstream cascades of immune response [10]. HMGB1 has been investigated as a biomarker for tumor outcome and therapy response in tumor tissue as well as in blood plasma or serum samples [11]. High HMGB1 expression was described as a negative prognostic factor in a meta-analysis of clinical studies [12]. In glioblastoma, recurrent tumors showed lower HMGB1 expression compared to the respective primary tumors [13]. However, the biology of intracellular HMGB1 in the tumor (as detected by immunohistochemistry in the tumor tissue) is different from the role of extracellular HMGB1, which mainly serves as a danger signal for the immune system. HMGB1 concentration in blood serum might rather be associated with this function of the protein and it has been described to be associated with prognosis in several cancer entities [14–16] and might serve as a biomarker in oncolytic virotherapy [17].

In cancer treatment, tumor-specific immune responses can be triggered by radiation [18] and by certain chemotherapeutics [19] as well as other forms of oncologic treatment such as locoregional hyperthermia [20, 21]. One mechanism described is immunogenic cell death (ICD), characterized by the release of danger signals (damage-associated molecular patterns, DAMPs) stimulating innate immune responses. HMGB1 has been described as one of the key players in ICD signaling in anticancer treatment [8] and as a consensus marker to monitor ICD in serum samples [22]. Of the markers of immunogenic cell death described in the Consensus guideline in 2015 [22], only HMGB1 can be measured in blood serum and plasma, as adenosine triphosphate (ATP) acts in the tumor microenvironment and calreticulin is exposed on tumor cells. In the updated guideline [23], other danger molecules have been added as possible ICD markers measurable in serum and plasma, such as the heat shock proteins (HSP) Hsp70 [24, 25] and Hsp90 [26]. In addition, correlation of serum markers with cellular immunomonitoring during radio(chemo)therapy [27, 28] might lead to further insights into systemic immune changes induced by cancer treatments.

However, besides being involved in ICD in cancer, HMGB1 also mediates diverse responses to inflammatory and infectious diseases [29, 30] such as, for example, pancreatitis and sepsis [31], pneumonia [32], stroke [33], or vasculitis [34]. This consideration might be important when monitoring ICD by HMGB1 during cancer therapy if inflammation and/or infection accompany the treatment.

Our intent in this study was to monitor HMBG1 during the course of radiochemotherapy in HNSCC patients and to correlate the dynamics with clinical features and outcome parameters.

## Material and methods

This prospective pilot biomarker study included 11 patients with newly diagnosed, locally advanced HNSCC. All patients provided written informed consent and the study was approved by the local ethics committee (reference number 064/2016BO2).

After exclusion of distant metastases, all patients were treated by intensity-modulated radiotherapy (IMRT) up to 70 Gy and concomitant chemotherapy with cisplatin weekly (*n* = 8) or 5‑flourouracil and mitomycin C (*n* = 3). Laboratory values, clinical features such as the gross tumor volume (GTV) of the primary tumors and the lymph nodes, as well as treatment-associated toxicity according to the Radiation Therapy Oncology Group (RTOG) grading were recorded. Furthermore, we recorded infections during therapy (defined by clinically manifest symptoms *plus* either antibiotic treatment or positive microbiological findings).

To monitor the dynamics of immune markers in the plasma, blood was taken before therapy on day 1 (*n* = 10) or on day 2 (*n* = 1) for baseline assessment and weekly thereafter. Routine blood samples were taken at the same time, e.g., to monitor blood count and C‑reactive protein (CRP) levels. To reduce potentially treatment-associated confounders, we took the weekly blood samples on Mondays (after the weekend) before irradiation or chemotherapy onset. “End of treatment” values are defined as the last available values during radiochemotherapy (range: day 28–46; mean: day 36 of treatment). If available, further samples and clinical data were collected during follow-up (3–6 monthly).

The blood collected in EDTA tubes (Sarstedt, Nümbrecht, Germany) was centrifuged twice for 10 min for plasma isolation and the plasma samples were stored at −80 °C. Samples were thawed immediately before use for HMGB1 analysis. All time points of individual patients were measured in one assay.

All available plasma samples were analyzed by enzyme-linked immunosorbent assays (ELISAs) to measure levels of HMGB1. The measurements were performed according to the manufacturer’s instructions (IBL International GmbH, Hamburg, Germany, Reference Number ST51011). This ELISA kit is specifically designed to measure HMGB1 in human serum and plasma in addition to cell culture medium. Standard curves were measured including HMGB1 concentrations of 0.625 ng/ml, 1.25 ng/ml, 2.5 ng/ml, 5.0 ng/ml, 10.0 ng/ml, 20.0 ng/ml, 40.0 ng/ml, and 80.0 ng/ml. The value of *R*^2^ for the fit of the standard curves was 0.99. Every sample was tested in technical duplicates. Means were used for analysis. For eight samples (9.4%), the duplicates showed limited reproducibility. However, for further analysis, all time points were used because all values were in a plausible range.

### Statistical analysis

Statistical analysis was performed with IBM SPSS Version 26 and GraphPad Version 8. Means were compared by the Mann–Whitney test if values did not pass the normality test, otherwise they were compared with Student’s *t* test. Bonferroni correction was applied in the case of multiple testing. Survival times were estimated with the Kaplan–Meier method and compared by log-rank-test. Correlations of continuous variables are described using Pearson correlation coefficients (*r*; moderate correlation defined as 0.4–0.7; strong correlation defined as > 0.7).

## Results

Eight men and three women were included in this study and all completed definitive radiochemotherapy. The patients’ characteristics are shown in Table 1**.** In total, 85 samples were collected (of these 61 during radiochemotherapy and 24 during follow-up). Overall, 11 planned samples during radiochemotherapy could not be collected or analyzed (e.g., because of acute infection or anemia of the patients or technical difficulties). Five samples were taken either after radiation or on Tuesdays before radiation treatment.

**Table 1:** Patients’ characteristics

–	*n*	%
**Gender**		
Female	3	27%
Male	8	73%
**Localization **		
Oropharynx	4	36%
Oropharynx/hypopharynx	1	9%
Hypopharynx	5	46%
Larynx	1	9%
**Human papillomavirus (HPV) status**		
Positive	5	46%
Negative	3	27%
Unknown	3	27%
**Involved lymph nodes**		
Yes	10	91%
No	1	9%

The follow-up ranged from 940 to 1476 days (mean = 1345). All but one patient received frequent clinical examinations and imaging for follow-up. This patient did not show up for further follow-up after 136 days but confirmed subjective well-being in a telephone interview after 1475 days. Nevertheless, for recurrence analysis, the short follow-up (136 days) was used.

**Fig. 1:**
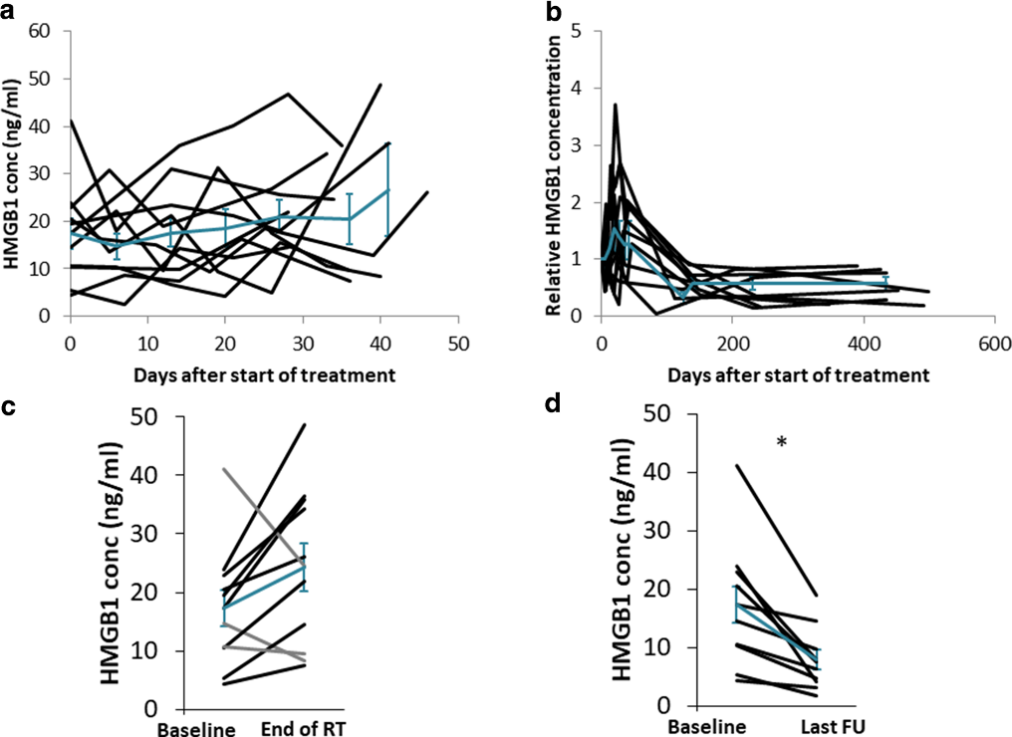
High Mobility Group Box 1 (HMGB1) concentrations were measured during radiochemotherapy (*RT*) as well as during follow-up (*FU*). While there were no consistent changes during treatment (**a**), HMGB1 levels decreased during follow-up in all patients (**b**). Comparing HMGB1 concentrations at baseline versus the last available samples during treatment, in eight patients concentrations increased, while three patients showed decreasing HMGB1 levels, indicated in *gray* (**c**). However, all HMGB1 concentrations at last follow-up were lower than initial levels (**d**). *Cyan* *lines* indicate means and standard errors for all patients

**Fig. 2:**
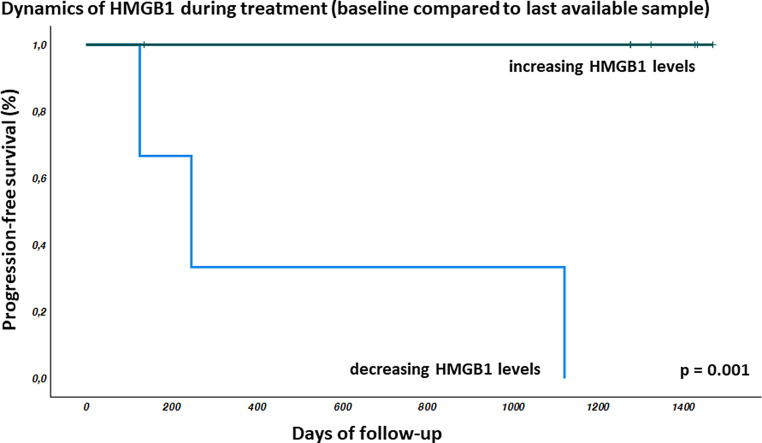
Three patients developed tumor recurrence during follow-up. Whereas there were no clinical features significantly influencing progression-free survival (data not shown), decreasing HMGB1 levels (end of treatment compared to baseline) significantly correlated with worse disease-free survival

In the consecutive sampling, HMGB1 showed undulating values during treatment (Fig. 1a). Relative HMGB1 concentrations normalized to the start of treatment intra-individually and follow-up values are shown in Fig. 1b. We compared the baseline value with end of treatment for each patient. In eight of 11 patients, the values were increasing toward the end of therapy (Fig. 1c). None of these patients showed tumor recurrence during follow-up. In three patients, HMGB1 levels in the last sample during radiochemotherapy were lower than the respective baseline. In all three patients with decreasing HMGB1, follow-up revealed tumor recurrence (Fig. 2; *p* = 0.001). Solitary lung metastases were treated (and histologically proven) by resection in two patients. One patient developed local recurrence and multiple metastases during follow-up. Comparing initial HMGB1 levels with change during therapy revealed a moderate negative correlation (*r* = −0.46; data not shown). Compared to the baseline values, the samples analyzed during follow-up showed lower levels of HMGB1 in all patients analyzed (*n* = 10; one patient did not show up for follow-up sampling) at all times (Fig. 1d).

**Fig. 3:**
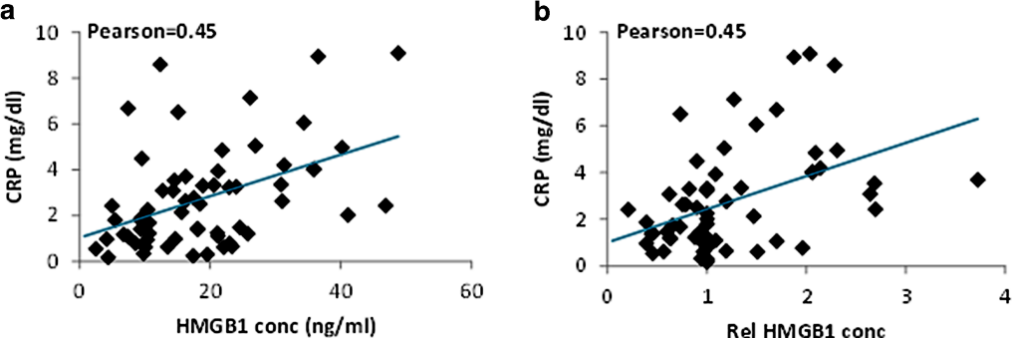
The inflammation marker C‑reactive protein (*CRP*) was correlated with the respective HMGB1 levels in a pooled analysis of all available time points during treatment. Both absolute HMGB1 concentrations (**a**) as well as relative HMGB1 levels normalized to baseline (**b**) showed a moderate correlation with CRP concentrations

**Fig. 4:**
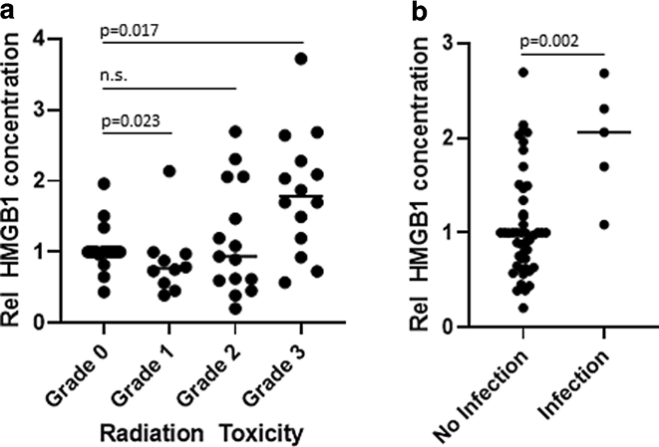
HMGB1 levels at all time points were normalized to the baseline value of the respective patient and correlated with Radiation Therapy Oncology Group(RTOG)-graded treatment toxicity as well as apparent infections. While patients with very mild toxicity showed a slight but significant decrease in HMGB1 levels, grade 3 toxicity was associated with a significant HMGB1 increase compared to time points at which patients did not show any treatment toxicity (**a**). Although infections were only present at five time points in four patients, they were associated with significantly higher relative HMGB1 concentrations compared to time points without apparent infections (**b**)

Since HMGB1 was found to be elevated in inflammatory and infectious diseases as well, we recorded corresponding CRP levels (*n* = 61) and data on infections (*n* = 53) and treatment-associated toxicity (*n* = 58) during therapy. HMGB1 levels were compared with inflammation and infection in a pooled analysis of all available data for all patients during treatment. HMGB1 showed a moderate positive correlation with CRP levels (Fig. 3; *r* = 0.45 for both, relative and absolute HMGB1 levels). Furthermore, time points with relevant radiation toxicity (grade 3 according to RTOG) showed significantly higher relative HMGB1 levels compared to time points without toxicity using a Bonferroni-corrected Mann–Whitney test (Fig. 4a, *p* = 0.017). Five samples were taken when a clinically manifest infection occurred at the same time. At time points with infections, mean HMGB1 levels were also significantly higher than in samples taken without simultaneous infections (Fig. 4b, *p* = 0.002). The CRP levels were not significantly higher at time points with manifest infections (3.5 ± 0.7 mg/dl vs. 2.6 ± 0.3 mg/dl, *p* = 0.37). Whereas there was no difference in CRP levels with grade 1 and grade 2 toxicity (data not shown), grade 3 toxicity was associated with significantly higher CRP levels compared to time points without toxicity (4.7 ± 0.8 mg/dl vs. 1.6 ± 0.3 mg/dl, *p* < 0.001).

**Fig. 5:**
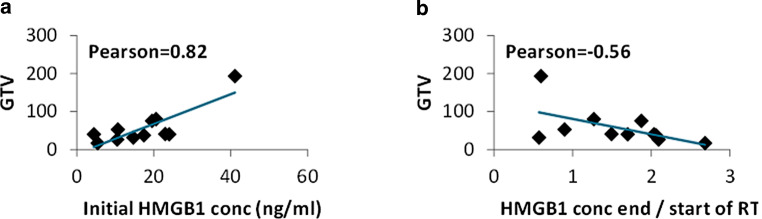
Contoured volumes retrieved from the radiotherapy plans (gross tumor volume [*GTV*] of the primary tumor and involved lymph nodes) were correlated with HMGB1 concentrations. GTV volumes showed a strong positive correlation with initial HMGB1 levels (**a**). Change in HMGB1 concentrations during treatment (ratio of the last available HMGB1 concentrations during treatment to baseline) showed moderate negative correlations with the GTV volume (**b**)

HMGB1 levels at time point 1 (baseline) were strongly correlated with the initial GTV (tumor + involved lymph nodes; *r* = 0.82) contoured for radiotherapy planning, as shown in Fig. 5a. After exclusion of the patient with the largest tumor volume, HMGB1 at baseline was moderately correlated with the GTV (*r* = 0.48). When analyzing the dynamics of HMGB1 levels during treatment (ratio of the last available sample to baseline), a moderate negative correlation with the GTV was observed (Fig. 5b).

## Discussion

HMGB1 was described to be a consensus marker for ICD [22]. Compared to healthy donors, HMGB1 levels seem to be elevated in the serum of HNSCC patients [35]. However, data on monitoring HMGB1 in patients during the course of curative radio(chemo)therapy are rare. In a report of four patients receiving definitive radiochemotherapy for HNSCC and 13 patients treated in an adjuvant intention, elevated HMGB1 levels were described during follow-up in cases of recurrence. However, the authors indicate a low specificity. In this study, HMGB1 was measured only once during radiotherapy [36]. To further evaluate the potential of this biomarker in patients with HNSCC, we investigated HMGB1 in weekly sampling during definitive radiochemotherapy and during follow-up in a homogenous cohort of patients undergoing curative definitive radiochemotherapy for locally advanced HNSCC.

In most patients, we found increasing HMGB1 levels when comparing start to end of treatment, with undulating concentrations during therapy, which might potentially reflect not only ICD levels but also diverse confounders such as inflammation and infection. Even though we only describe dynamics in a small cohort, it is remarkable that we found a correlation with oncological outcome parameters. None of the eight patients with increasing HMGB1 levels during therapy experienced tumor recurrence according to our follow-up data, while all three patients with declining HMGB1 levels showed local and/or distant treatment failure. These findings are in line with a report about patients with esophageal cancer treated with radiochemotherapy [37]. In this study, HMBG1 was measured in serum before treatment and within 3 days of the end of treatment. Elevated HMGB1 levels were found at the end of therapy. Patients with tumor antigen-specific T‑cell response showed significantly higher levels of HMGB1 at the end of treatment compared to patients without this specific T‑cell response, probably indicating better anti-tumor immune responses possibly related to better tumor control.

Interestingly, in our cohort, two of the three patients with tumor relapse suffered from distant metastases while the tumor was locally controlled. One could speculate that in these patients the radiation dose was sufficient for local control but distant lesions occurred due to impaired systemic immune response associated with reduced ICD compared to the patients with rising HMGB1 levels during treatment.

Although our pilot results need to be confirmed in larger studies, the initial findings are very promising in regard to monitoring ICD and tumor response during treatment. If our results can be confirmed in the future, personalized treatment adaptions like (de)escalation could be discussed or follow-up intervals might be adapted and/or the addition of immunotherapeutic options according to immune status might be discussed.

However, especially in treatments with severe side effects and treatment-associated toxicities, confounders need to be considered when attributing rising HMGB1 levels to ICD in tumors. Radiochemotherapy in HNSCC causes dermatitis and mucositis as expected side effects. Furthermore, patients are immunocompromised due to concomitant chemotherapy and are prone to infections such as, for example, pneumonia or inflamed feeding tubes. In our cohort, manifest infections were rare (five time points). Thus, in spite of the significant correlation of infections and elevated HMGB1 levels, this sample size is too small for strong conclusions. However, in the literature [31, 32] the role of HMGB1 in inflammatory and infectious diseases was described before in different settings. Therefore, we believe, infections and high-grade toxicity should be reported and considered as potential confounders. HMGB1 levels at these time points might not reflect solely ICD and should be interpreted cautiously.

Furthermore, we found an association of elevated HMGB1 levels with rising CRP levels and with RTOG grade 3 toxicity. This is reasonable, as a positive correlation between CRP levels and HMGB1 was described before (in the setting of autoimmunity and inflammation; [31, 34]). As toxicity and inflammation are commonly elevated toward the end of treatment, it is hard to discriminate these confounders from ICD if rising HMGB1 levels are detected during therapy. This finding needs to be critically addressed and monitored if HMGB1 is used as a biomarker of ICD and linked to tumor response in future studies. Thus, we suggest special attention is given to treatment-associated confounders if severe side effects are expected such as toxicity ≥ 3 (RTOG grading) or whenever a patient has significantly rising CRP levels and manifest infections. Perhaps HMGB1 is an even more promising marker in particular types of cancer where no extensive toxicity is to be expected during radiotherapy.

The baseline levels of HMGB1 showed a positive correlation with the initial tumor volume, and during follow-up, all HMGB1 levels were lower than the respective baseline values. As only one patient had a significant tumor load at recurrence, we consider these findings promising. HMGB1 might reflect tumor necrosis pretherapeutically and it potentially qualifies as a follow-up marker.

## Conclusion

In our longitudinal pilot study, High Mobility Group Box 1 protein (HMGB1) was found to be a promising biomarker to monitor tumor response in definitive radiochemotherapy for head and neck squamous cell carcinoma. However, besides outcome data, correlations with inflammation, toxicity, and infection could also be seen. Therefore, potential confounders must be considered when monitoring and interpreting HMGB1 levels during cancer treatment.
